# Microbiota-friendly diet ameliorates hypoalbuminemia in chronic kidney disease: evidence from NHANES

**DOI:** 10.3389/fimmu.2025.1546031

**Published:** 2025-05-06

**Authors:** Xiaoyan Wang, Pengfei Wen, Fang Gao, JinXiu Zhao, Shuchuan Miao

**Affiliations:** ^1^ Department of Clinical Nutrition, The First Affiliated Hospital of Chengdu Medical College, Chengdu, Sichuan, China; ^2^ Department of Dermatology, West China Hospital, Sichuan University, Chengdu, China; ^3^ Department of Nephrology, The First Affiliated Hospital of Chengdu Medical College, Chengdu, Sichuan, China; ^4^ Department of Vascular Surgery, The First Affiliated Hospital of Chengdu Medical College, Chengdu, Sichuan, China; ^5^ Department of Neurosurgery, Hospital of Chengdu University of Traditional Chinese Medicine, Chengdu, Sichuan, China

**Keywords:** CKD, dietary index for gut microbiota, serum albumin, hypoalbuminemia, dietary pattern

## Abstract

Chronic kidney disease (CKD) is a global health issue, affecting approximately 10% of the population. Hypoalbuminemia, a common complication in advanced CKD, is associated with poor prognosis. This study aimed to investigate the association between a microbiota-friendly dietary scoring system (Dietary Index for Gut Microbiota, DI-GM) and serum albumin levels in patients with CKD. We utilized a cross-sectional cohort from the NHANES 2007–2018, which included 2,947 CKD patients. Multivariable logistic regression and restricted cubic spline models were applied to analyze the relationship between DI-GM scores and serum albumin. Higher DI-GM scores were significantly associated with increased serum albumin levels (β = 0.18 g/L, 95% CI: 0.07–0.28, p = 0.002). Furthermore, each 1-point increase in DI-GM score was linked to a 15% reduction in the odds of hypoalbuminemia (OR: 0.85, 95% CI: 0.74–0.97, p = 0.014). The findings suggest that a high DI-GM diet may have beneficial effects in managing hypoalbuminemia in CKD patients by modulating gut microbiota composition and reducing inflammation. This diet pattern could be a promising dietary intervention for improving clinical outcomes in CKD patients, especially those at risk for malnutrition and inflammation.

## Introduction

Chronic kidney disease (CKD) has emerged as a significant global health issue, affecting approximately 10% of the population worldwide, with cases spanning early to advanced stages of the disease, ranging from early to advanced stages of the disease (1, 2).One common complication in CKD is hypoalbuminemia, characterized by low serum albumin levels, which affects about 30% to 50% of patients, especially those in advanced stages (3). Albumin, the most abundant protein in blood plasma, plays an essential role in maintaining colloidal osmotic pressure and antioxidant capacity (4, 5). It also serves as an indicator of chronic inflammation and protein-energy wasting (PEW), a condition that poses additional health risks for CKD patients (6).

The underlying causes of hypoalbuminemia are multifactorial and complex, often involving reduced protein synthesis, increased catabolism, oxidative stress, and nutritional deficiencies (7–9). Research has shown that CKD patients frequently experience significant shifts in gut microbiota, marked by a reduction in beneficial bacteria and an increase in pathogenic species (10–14), contributing to systemic oxidative stress, elevated inflammation (15, 16), and an exacerbation of hypoalbuminemia (4). Furthermore, dietary modifications in CKD, such as low-protein diets and restrictions on fruits and vegetables, may further disrupt gut microbiota (17, 18), perpetuating a vicious cycle of microbial imbalance and inflammation.

Given these concerns, optimizing gut microbiota composition has emerged as a potential therapeutic strategy for addressing hypoalbuminemia in CKD. Dietary management has become critical in this context. The Dietary Index for Gut Microbiota (DI-GM) is a novel dietary assessment tool developed to evaluate diet’s impact on gut health by measuring microbial diversity and short-chain fatty acid production. This index considers the intake of 14 food items, both beneficial (e.g., fermented dairy products, whole grains) and detrimental (e.g., red and processed meats).Studies have positively correlated DI-GM scores with biomarkers indicating gut microbiota diversity, supporting its validity and applicability (19).

Based on existing literature, we hypothesize that the regulation of gut microbiota by the Dietary Index (DI-GM) may be associated with improved serum albumin levels in CKD patients. This study aims to systematically analyze the association between DI-GM and serum albumin levels in CKD patients and explore potential underlying mechanisms. By examining the impact of this dietary index, we hope to provide new evidence for future gut microbiota-related interventions in CKD management.

## Materials and methods

### Participants

This cross-sectional study utilized data from the NHANES from 2007 to 2018, which was approved by the Institutional Review Board of the National Center for Health Statistics. All participants provided written informed consent, and the use of de-identified public data exempted the study from further ethical review. The study adheres to the Strengthening the Reporting of Observational Studies in Epidemiology (STROBE) guidelines.

**Figure 1:**
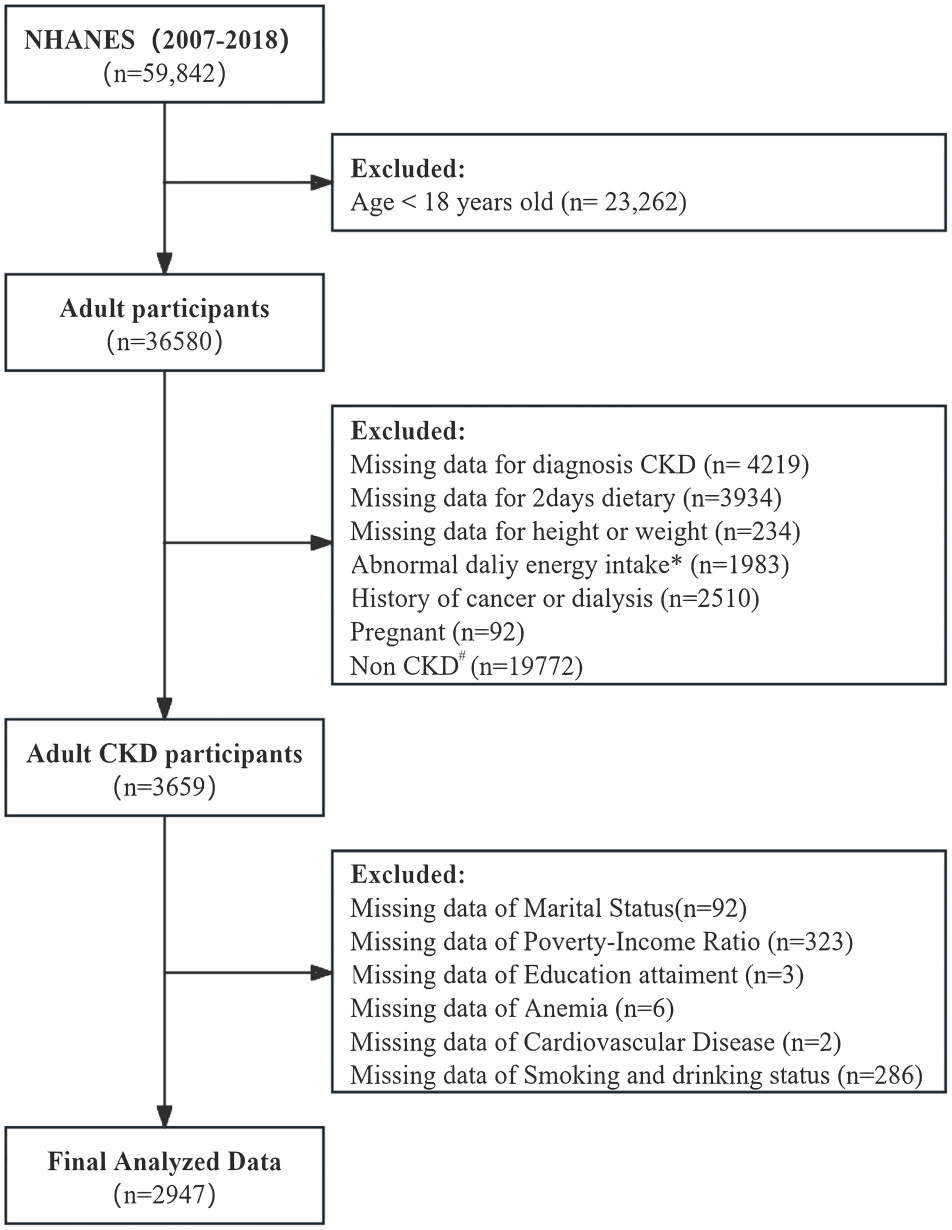
Study Population and flowchart. #: Diagnosis of chronic kidney disease (CKD): estimated glomerular filtration rate (eGFR) <60 mL/min/1.73 m² or urinary albumin-to-creatinine ratio (UACR) ≥30 mg/g. Complete and reliable dietary intake data for calculating DI-GM. *Abnormal dietary records: <800 kcal/day or >4000 kcal/day for men; <500 kcal/day or >3500 kcal/day for women.

We included NHANES participants aged 18 years and older (n=36580). Exclusions were applied for participants with missing values to calculated albumin-to-creatinine ratio (ACR) and estimated glomerular filtration rate (eGFR) data. Those lacking two-day dietary data, missing height or weight data, abnormal energy intake (<800 or >4000 kcal/day for men; <500 or >3500 kcal/day for women), and individuals with a history of cancer or dialysis, pregnant and not meeting the diagnostic criteria for CKD, we also excluded the missing covariate data. The final analytic cohort consisted of 2947 individuals, representing 19,690,044.94 U.S. CKD adults (**Figure 1**). Sociodemographic variables, lifestyle variables, laboratory variables and dietary data were collected.

### Definition

CKD classification was based on a urinary albumin-to-creatinine ratio (≥30 mg/g or 3 mg/mmol) or estimated glomerular filtration rate (eGFR) <60 mL/min/1.73 m² (20). eGFR was calculated using the CKD-EPI- creatinine formula. Given that nutritional interventions typically start at stage G3 (21), we classified G1(eGFR ≥ 90 mL/min/1.73 m²)-G2(eGFR 60–89 mL/min/1.73 m²) as early CKD and G3a-G5(G3a: eGFR 45–59 mL/min/1.73 m², G3b: eGFR 30–44 mL/min/1.73 m², G4: eGFR 15–29 mL/min/1.73 m², G5:eGFR < 15 mL/min/1.73 m²) as advanced CKD. Hypoalbuminemia is defined as a serum albumin level less than 38 g/L (6).

### Dietary assessment

Dietary data from NHANES was collected through two 24-hour recalls, spaced 3–10 days, utilizing the USDA’s Automated Multiple-Pass Method. These recalls were conducted at MECs and via telephone, respectively. Standardized tools aided in portion size estimation. The USDA’s FNDDS was employed to code all foods and beverages and their amounts (22). The DI-GM was then constructed using the averaged intake from the two recalls.

### DI-GM

To quantify dietary influence on gut microbiota, a scoring system was developed (19). 14 foods or nutrients were identified as components of the DI-GM, including fermented dairy, chickpeas, soybean, whole grains, fiber, cranberries, avocados, broccoli, coffee, and green tea as beneficial components, and red meat, processed meat, refined grains, and high-fat diet (≥40% of energy from fat) as unfavorable components. Each component was scored 0 or 1 based on sex-specific median intakes, participants who met the criteria for healthy intake (above the sex-specific median for beneficial components, or below for unfavorable components) received a score of 1. Those who did not meet these criteria received a 0. The aggregate of these scores produced the DI-GM score, a measure from 0 to 14, where higher values correlate with enhanced gut microbiota health (**Supplementary Table S1**).

In this study, we divided DI-GM into tertiles based on score ranges: the first tertile, T1(DI-GM scores 0-3, n=600), the second tertile, T2 (DI-GM scores 4-5, n=1331), and the third tertile, T3 (DI-GM scores 6-10, n=1061).

### Covariates

Covariates were selected based on existing literature and clinical judgment. Binary logistic regression was used to identify confounders that altered the initial coefficients by more 10%. Multicollinearity was assessed with the variance inflation factor (VIF), with VIF values ≥5 indicating multicollinearity. The final covariates included age, sex, poverty-income ratio (PIR), Education attainment, marital status, body mass index (BMI), smoking and drinking status, hyperlipidemia history, eGFR, urinary albumin, energy intake (kcal/kg/day), and protein intake (g/kg/day), cardiovascular disease (CVD), hyperlipidemia, hypertension and diabetes mellitus (DM).

### Statistical analyses

Two-day dietary weights, adjusted for the six survey cycles, were applied to represent the U.S. population aged 18 years and older, accounting for the complex survey design and non-response bias.

Descriptive statistics were used to summarize baseline characteristics, nutrient intake, dietary quality, and frailty incidence across different survey years. Continuous variables were presented as mean ± standard error (SE) for normal distributed data, or as median and interquartile range (IQR) for skewed data, while categorical variables were reported as frequencies and percentages.

Weighted univariable and multivariable logistic regression models were employed to assess the association between DI-GM and serum albumin/hypoalbuminemia across CKD stages, with weighted odds ratios (ORs) and 95% confidence intervals (CIs) calculated before and after adjusting for confounders. Three models were developed to adjusted for (1): age, sex, race (2); age, sex, race, education, marital status, PIR, BMI, smoking status, drinking status; and (3) adding hyperlipidemia, CVD, hypertension, DM, eGFR, urinary albumin, energy, and protein daily intake (adjusted by standard body weight).

A weighted Restricted Cubic Spline (RCS) model with three knots examined the potential non-linear relationship between DI-GM and serum albumin, with 1,000 bootstrap replications for robustness. Subgroup analyses stratified by age, sex, BMI, smoking, CKD stage, diabetes, energy, and protein intake were conducted using multivariable logistic regression to calculate ORs (95% CI). Results were visualized using a forest plot.

We also conducted the further univariable and multivariable analysis of components’ score in the DI-GM and their association with serum albumin levels to find the most important details among the components. We found the unfavorable components score of refined grains was the most related factor, moreover, we further explore the association between refined grain intake daily and serum albumin level with univariable and multivariable logistic regression analysis.

All analyses used weighted data, with statistical significance was set at p<0.05. Statistical analyses were conducted using R (v4.4.1) and Free Statistics software (version 1.9.2; Beijing Free Clinical Medical Technology Co., Ltd.).

## Results

A total of 2,947 participants, representing an estimated 19.60 million adults with CKD in the US, were included (**Figure 1**, **Table 1**). Baseline characteristics of participants are shown in **Table 1**. The mean age was 58.21 years, and 40.25% were male. Significant sociodemographic differences were observed across DI-GM tertiles. Non-Hispanic Black individuals comprised 18.01% of the lowest tertile, while Non-Hispanic White individuals accounted for 74.51% of the highest tertile (P < 0.001). Higher education levels were associated with the highest tertile, with 63.89% being college-educated (P <0.001), and the PIR was significantly lower in the lowest tertile (P < 0.001). Regarding lifestyle factors, smoking prevalence was highest in the middle tertile (20.05%) and lowest in the highest tertile (13.47%; P =0.013). For clinical and laboratory variables, albumin levels were highest in the top tertile (42.21 g/L in T3; P = 0.001), whereas serum creatinine and uric acid levels were significantly elevated in the lowest tertile (P = 0.029 and P = 0.015, respectively). Serum bicarbonate levels were significantly lower in the lowest tertile (P =0.003). No significant differences in comorbidity incidence were found among the three tertiles (**Table 1**).

**Table 1:** Baseline characteristics of adults with CKD from NHANES 2007-2018.

Variables	Overall	Dietary Index for Gut Microbiota			P value
		T1[0-3]	T2[4-5]	T3[6-10]	
Unweighted Number	2947	600	1331	1016	
Weighted Number	19690044.94	3598766.6	8131071.29	7960207.05	
Sociodemographic variables					
Age (year)	58.21 (16.80)	57.19 (17.33)	58.05 (16.91)	58.82 (16.43)	0.376
Sex (Male) (%)	40.25	42.77	41.23	38.1	0.38
Race (%)					<0.001
Mexican American	7.7	7.84	8.83	6.47	
Non-Hispanic Black	11.69	18.01	12.69	7.82	
Non-Hispanic White	68.98	62.38	66.48	74.51	
Other race	11.63	11.77	12	11.19	
Marital Status (%)					0.296
Living with partner	61.18	56.33	63.21	61.30	
Living alone	38.82	43.67	36.79	38.70	
Education (%)					<0.001
College	53.52	40.32	49.19	63.89	
High School	26.1	32.77	26.83	22.34	
Less than high school	20.38	26.9	23.97	13.76	
Poverty-Income Ratio	2.79 (1.66)	2.46 (1.59)	2.63 (1.64)	3.09 (1.66)	<0.001
Body Mass Index (kg/m^2^)	30.68 (7.64)	31.56 (8.62)	31.23 (7.64)	29.73 (7.04)	0.005
Lifestyle variables					
Smoking (Yes) (%)	16.61	15.79	20.05	13.47	0.013
Drinking (Yes) (%)	66.38	62.69	66.19	68.25	0.275
Clinical variables					
Chronic Kidney Disease (CKD) (%)					0.952
Early CKD	56.60	55.68	56.61	57.01	
Advanced CKD	43.40	44.32	43.39	42.99	
Cardiovascular Disease (Yes) (%)	21.2	20.81	22.01	20.56	0.81
Hypertension (Yes) (%)	64.54	63.81	67.29	62.06	0.25
Hyperlipidemia (Yes) (%)	82.63	85.61	82.75	81.16	0.378
Anemia (Yes) (%)	12.91	16.57	12.72	11.45	0.098
Diabetes Mellitus (Yes) (%)	34.5	38.58	36.03	31.09	0.099
Laboratory variables					
Albumin (g/L)	41.86 (3.33)	41.17 (3.56)	41.83 (3.22)	42.21 (3.29)	0.001
Hypoalbuminemia (%)					0.013
Albumin<38(g/L)	7.46	10.88	7.95	5.42	
Albumin≥38(g/L)	92.54	89.12	92.05	94.58	
Serum Creatinine (umol/L)	91.23 (35.13)	96.38 (40.89)	90.39 (33.32)	89.75 (33.92)	0.029
Blood Urea Nitrogen (mmol/L)	6.04 (2.92)	6.32 (3.39)	5.99 (2.96)	5.97 (2.62)	0.252
eGFR (ml/min/1.73 m^2^)	76.50 (28.39)	75.24 (29.55)	77.37 (28.79)	76.19 (27.43)	0.572
Urinary Albumin (mg/L)	163.23 (456.98)	193.36 (517.04)	160.71 (384.07)	152.18 (494.85)	0.431
Urinary Creatinine (mg/L)	9770.29 (6468.87)	10438.91 (6133.73)	9965.19 (6566.06)	9268.92 (6483.90)	0.051
Uric Acid (umol/L)	354.56 (99.32)	369.30 (103.65)	356.97 (96.11)	345.45 (99.70)	0.015
Serum Sodium (mmol/L)	139.21 (2.69)	139.24 (2.79)	139.08 (2.77)	139.32 (2.55)	0.342
Serum Potassium (mmol/L)	4.08 (0.41)	4.09 (0.48)	4.06 (0.40)	4.09 (0.39)	0.393
Serum Phosphorus (mmol/L)	1.21 (0.18)	1.19 (0.19)	1.21 (0.18)	1.22 (0.18)	0.079
Serum Iron (umol/L)	14.63 (5.86)	14.36 (6.18)	14.69 (5.86)	14.69 (5.70)	0.802
Serum Calcium (mmol/L)	2.31 (0.10)	2.32 (0.10)	2.31 (0.10)	2.31 (0.10)	0.555
Serum Bicarbonate (mmol/L)	25.04 (2.41)	24.72 (2.52)	24.90 (2.35)	25.32 (2.39)	0.003

Continuous variables: mean ± standard deviation (SD), Categorical variables: frequencies and percentages.

eGFR, estimated glomerular filtration rate.

### Dietary data and components of DI-GM

Dietary intake and food group consumption across tertiles of the Dietary Index for Gut Microbiota (DI-GM) in participants with CKD are summarized in **Table 2**. Energy and protein intake did not differ significantly across tertiles. However, carbohydrate intake increased progressively from the lowest to the highest tertile (P < 0.001), and fiber intake was significantly higher in the highest tertile compared with the other tertiles (P <0.001). Total fat intake and saturated fat intake were highest in the lowest tertile (P = 0.036 and P =0.044, respectively). As DI-GM scores increased, the proportion of individuals with a score of 1 for specific food items also rose (P < 0.01 for all). Scores for all DI-GM components, including avocado, broccoli, chickpea, coffee, cranberry, fermented dairy, fiber, green tea, soybean, whole grains, refined grains, processed meat, and fat, increased significantly across higher DI-GM tertiles (P < 0.001 for all).

**Table 2:** Nutrient and food group daily intake across tertiles of the dietary index for gut microbiota in CKD patients.

Variables	Overall	Dietary Index for Gut Microbiota			P value
		T1[0-3]	T2[4-5]	T3[6-10]	
Unweighted Number	2947	600	1331	1016	
Weighted Number	19690044.94	3598766.6	8131071.29	7960207.05	
Energy intake (kcal/d)	1884.79 (717.01)	1826.47 (678.38)	1865.93 (754.08)	1930.42 (692.56)	0.221
Energy intake (kcal/kg/d)	31.09 (11.19)	30.35 (10.86)	30.85 (11.77)	31.66 (10.70)	0.387
Protein intake (g/d)	73.82 (33.28)	73.35 (31.25)	72.71 (36.68)	75.17 (30.37)	0.469
Protein intake (g/kg/d)	1.22 (0.52)	1.22 (0.52)	1.20 (0.57)	1.23 (0.48)	0.675
Protein-to-Energy Ratio	0.16 (0.05)	0.17 (0.05)	0.16 (0.05)	0.16 (0.05)	0.194
Carbohydrate (g/d)	223.86 (95.55)	194.09 (92.44)	217.26 (92.98)	244.06 (95.13)	<0.001
Carbohydrate-to-Energy Ratio	0.48 (0.11)	0.43 (0.12)	0.48 (0.12)	0.51 (0.10)	<0.001
Diet Fiber (g/d)	15.78 (9.22)	10.54 (6.42)	14.08 (8.21)	19.88 (9.53)	<0.001
Total Fat (g/d)	73.46 (36.68)	78.75 (36.33)	73.17 (40.60)	71.38 (32.14)	0.036
Total Fat -to-Energy Ratio	0.35 (0.09)	0.39 (0.10)	0.34 (0.09)	0.33 (0.08)	<0.001
SFA (g/d)	23.89 (13.19)	25.81 (13.18)	23.95 (13.86)	22.97 (12.39)	0.044
SFA -to-Energy Ratio	0.11 (0.04)	0.13 (0.04)	0.11 (0.04)	0.10 (0.04)	<0.001
MUFA (g/d)	26.08 (14.29)	28.94 (14.32)	25.89 (15.82)	24.98 (12.34)	0.002
MUFA -to-Energy Ratio	0.12 (0.04)	0.14 (0.04)	0.12 (0.04)	0.12 (0.04)	<0.001
PUFA(g/d)	16.87 (10.30)	16.96 (9.56)	16.67 (11.35)	17.02 (9.45)	0.878
PUFA -to-Energy Ratio	0.08 (0.03)	0.08 (0.04)	0.08 (0.04)	0.08 (0.03)	0.078
Dietary Index for Gut Microbiota	5.07 (1.72)	2.54 (0.66)	4.53 (0.50)	6.76 (0.92)	<0.001
Score of avocado = 1 (%)	3.73	0.34	1.81	7.24	<0.001
Score of broccoli = 1 (%)	15.66	4.73	9.89	26.5	<0.001
Score of chickpea = 1 (%)	0.98	0.07	0.3	2.09	0.001
Score of coffee = 1 (%)	39.36	19.66	36.05	51.65	<0.001
Score of cranberry = 1 (%)	8.52	5	5.71	12.99	<0.001
Score of fermented dairy = 1 (%)	45.02	24.49	46.12	53.17	<0.001
Score of fiber = 1 (%)	53.6	18.15	45.99	77.39	<0.001
Score of green tea = 1 (%)	20.35	8.74	17.1	28.92	<0.001
Score of soybean = 1 (%)	23.02	6.08	15.42	38.45	<0.001
Score of whole grains = 1 (%)	30.96	3.89	18.86	55.56	<0.001
Refined grains (oz.eq.)	5.01 (3.36)	5.07 (3.04)	5.20 (3.48)	4.78 (3.35)	0.231
Score of refined grains = 1 (%)	56.65	49.65	53.08	63.45	0.009
Score of processed meat = 1 (%)	74.34	40.54	74.12	89.84	<0.001
Score of fat = 1 (%)	73.45	46.08	73.15	86.14	<0.001

Continuous variables: mean ± standard deviation (SD), Categorical variables: frequencies and percentages.

SFA, Saturated fatty acids; MUFA, Monounsaturated fatty acids; PUFA, Polyunsaturated fatty acids.

### Association between total DI-GM, the components details and serum albumin levels

Weighted univariable and multivariable analyses showed that each 1-point increase in DI-GM score was significantly associated with higher serum albumin levels across all models. In fully adjusted Model 3, each 1-point increase in DI-GM corresponded to a 0.18 g/L increase in serum albumin (95% CI, 0.07-0.28; P =0.002). When analyzed by tertiles, participants in T2 (DI-GM 4-5) and T3 (DI-GM 6-10) had significantly higher serum albumin levels compared with T1 (DI-GM 0-3), with the most pronounced increase in T3 at 0.82 g/L in Model 3 (95% CI, 0.32-1.33; P = 0.002) (**Table 3**).

**Table 3:** Weighted univariable and multivariable analysis of the association between DI-GM and serum albumin levels in CKD patients.

Estimated DI-GM	Serum Albumin Levels (g/L)							
	Crude Model		Model 1		Model 2		Model 3	
	β(95%CI)	P value	β(95%CI)	P value	β(95%CI)	P value	β(95%CI)	P value
DI-GM (Continuous)	0.21(0.08, 0.34)	0.001	0.22(0.10, 0.34)	<0.001	0.17(0.06, 0.28)	0.004	0.18(0.07, 0.28)	0.002
DI-GM (Category)								
T1[0-3]	Reference		Reference		Reference		Reference	
T2[4-5]	0.66(0.11,1.20)	0.018	0.65(0.11, 1.20)	0.019	0.65(0.11, 1.19)	0.019	0.64(0.11, 1.17)	0.019
T3[6-10]	1.04(0.49,1.60)	<0.001	1.02(0.46, 1.59)	<0.001	0.79(0.28, 1.30)	0.003	0.82(0.32, 1.33)	0.002

Model 1: Adjusted for Age, sex and race.

Model 2: Adjusted for Model 1+ PIR+Education+Marital Status +BMI+Smoking status+Drinking status.

Model 3: Adjusted for Model 2 + eGFR+ urinary albumin +Energy intake(kcal/kg/d) +Protein intake(g/kg/d) + cardiovascular disease +Hyperlipidemia+Hypertension+ Diabetes Mellitus.

Unweighted/weighted Number of groups: DI-GM T1[0-3]: 600/3598766.6; DI-GM T2[4-5]: 1331/813071.29; DI-GM T3[6-10]: 1061/7960207; Total number: 2947/19690044.94.

For hypoalbuminemia (serum albumin < 38 g/L), weighted univariable and multivariable analyses indicated that each 1-point increase in DI-GM score was associated with a significant reduction in the odds of hypoalbuminemia across all models. In Model 3, each 1-point increase in DI-GM was associated with a 15% reduction in odds (OR, 0.85; 95% CI, 0.74-0.97; P = 0.014). Categorically, participants in T3 showed the strongest inverse association with hypoalbuminemia, with a 49% reduction in odds compared with T1 in Model 3 (OR, 0.51; 95% CI, 0.29-0.91; P =0.024) (**Table 4**).

**Table 4:** Weighted univariable and multivariable analysis of the association between DI-GM and hypoalbuminemia in CKD patients.

Estimated DI-GM	Hypoalbuminemia (ALB <38g/L)							
	Crude Model		Model 1		Model 2		Model 3	
	OR (95%CI)	P value	OR (95%CI)	P value	OR (95%CI)	P value	OR (95%CI)	P value
DI-GM (Continuous)	0.83(0.73, 0.94)	0.003	0.84(0.75, 0.95)	0.007	0.86(0.76, 0.97)	0.018	0.85(0.74, 0.97)	0.014
DI-GM (Category)								
T1[0-3]	Reference		Reference		Reference		Reference	
T2[4-5]	0.71(0.46, 1.10)	0.121	0.72(0.46, 1.14)	0.157	0.74(0.47, 1.16)	0.182	0.72(0.45, 1.15)	0.164
T3[6-10]	0.47(0.28, 0.80)	0.006	0.51(0.30, 0.88)	0.016	0.56(0.33, 0.95)	0.032	0.51(0.29, 0.91)	0.024

Model 1:Adjusted for Age, sex and race.

Model 2:Adjusted for Model 1+ PIR+Education+Marital Status +BMI+Smoking status+Drinking status.

Model 3:Adjusted for Model 2 + eGFR+ urinary albumin +Energy intake(kcal/kg/d) +Protein intake(g/kg/d) + cardiovascular disease +Hyperlipidemia+Hypertension+ Diabetes Mellitus.

Unweighted/weighted Number of groups: DI-GM T1[0-3]: 600/3598766.6; DI-GM T2[4-5]: 1331/813071.29; DI-GM T3[6-10]: 1061/7960207; Total number: 2947/19690044.94.

Weighted number of events: Total event: 1468877.35, DI-GM T1[0-3]: 391545.81, DI-GM T2[4-5]: 646420.17, DI-GM T3[6-10]: 431443.

The association between individual dietary components of the DI-GM and serum albumin levels showed, in the crude model, a score of 1 for refined grain intake was associated with a 0.56 g/L increase in serum albumin (β = 0.56, 95% CI: 0.13, 0.98, p = 0.01). This association remained significant in the adjusted model, with a 0.41 g/L increase in serum albumin (β = 0.41, 95% CI: 0.02, 0.80, p = 0.041). Regarding other components, in the crude model, only the intake of whole grains above the sex-specific median (i.e., a score of 1) was associated with a 0.51 g/L increase in serum albumin (β = 0.51, 95% CI: 0.02, 1.01, p = 0.042). However, in the adjusted model, this association was no longer significant (β = 0.41, 95% CI: -0.04, 0.85, p = 0.072). (**Supplementary Table S2**).

### Association between score of refined grain, refined grain intake and serum albumin levels

A nuanced association between refined grain intake and serum albumin levels in CKD patients. Got the score of refined grains (score = 1) was positively associated with serum albumin levels across all models, with an increase of 0.41 g/L in the fully adjusted model (95% CI: 0.02, 0.80, p = 0.041). However, when analyzed as a continuous variable (oz. eq.), increased daily refined grain intake was inversely associated with serum albumin levels, with a 0.07 g/L decrease per ounce equivalent in the fully adjusted model (95% CI: -0.13, -0.01, p = 0.032) (**Table 5**).

**Table 5:** Weighted univariable and multivariable analysis of the association between refined grains and serum albumin levels in CKD patients.

Estimated Refined grains score or intake	Serum Albumin Levels (g/L)							
	Crude Model		Model 1		Model 2		Model 3	
	β(95%CI)	P value	β(95%CI)	P value	β(95%CI)	P value	β(95%CI)	P value
Score of Refined grains								
0	Reference		Reference		Reference		Reference	
1	0.56(0.13,0.98)	0.01	0.66(0.27, 1.06)	0.001	0.52(0.14, 0.89)	0.007	0.41(0.02,0.80)	0.041
Refined grains intake (oz.eq.) (Continuous)	-0.06(-0.12, -0.01)	0.032	-0.11(-0.17, -0.06)	<0.001	-0.09(-0.14, -0.03)	0.002	-0.07(-0.13, -0.01)	0.032

Model 1:Adjusted for Age, sex and race.

Model 2:Adjusted for Model 1+ PIR+Education+Marital Status +BMI+Smoking status+Drinking status.

Model 3:Adjusted for Model 2 + eGFR+ urinary albumin +Energy intake(kcal/kg/d) +Protein intake(g/kg/d) + cardiovascular disease +Hyperlipidemia+Hypertension+ Diabetes Mellitus.

Unweighted/weighted Number of groups: DI-GM T1[0-3]: 600/3598766.6; DI-GM T2[4-5]: 1331/813071.29; DI-GM T3[6-10]: 1061/7960207; Total number: 2947/19690044.94.

### Sensitive analysis

We conducted RCS to find the nonlinear relationship between DI-GM, refined grains consumed and serum albumin level. As the DI-GM score increases beyond the reference point (DI-GM = 5), there is a significant increase in serum albumin levels.

**Figure 2:**
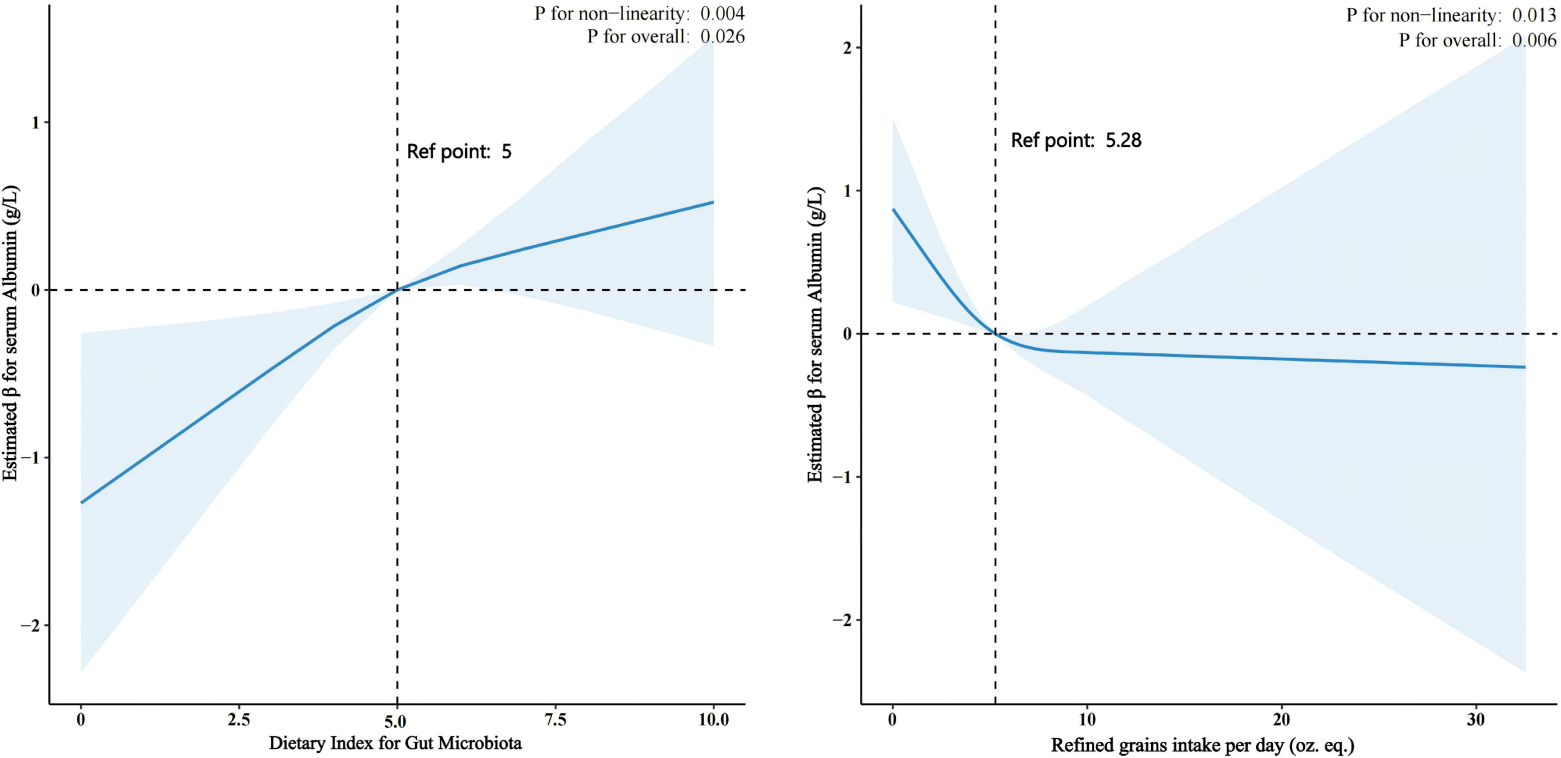
The Relationship Between Dietary Index for Gut Microbiota (DI-GM) and Refined Grains Intake with Serum Albumin Levels in CKD Patients Using Restricted Cubic Splines (RCS). DI-GM and Serum Albumin Levels: An RCS analysis revealed a nonlinear relationship (p = 0.004). Serum albumin levels significantly increase as the DI-GM score exceeds 5. Refined Grain Intake and Serum Albumin Levels: An RCS analysis showed a nonlinear relationship (p = 0.013). At approximately 5.28 oz. eq. of refined grains per day, albumin levels remain stable. However, intake beyond this point is associated with a decrease in serum albumin levels.

A non-linear relationship between refined grains intake and serum albumin levels (p for non-linearity = 0.013, p for overall = 0.006) was also found. At the reference point of approximately 5.28 oz. eq. of refined grains intake per day, albumin levels remain relatively stable. However, increasing intake beyond this point is associated with a decrease in serum albumin levels. (**Figure 2**).

**Figure 3:**
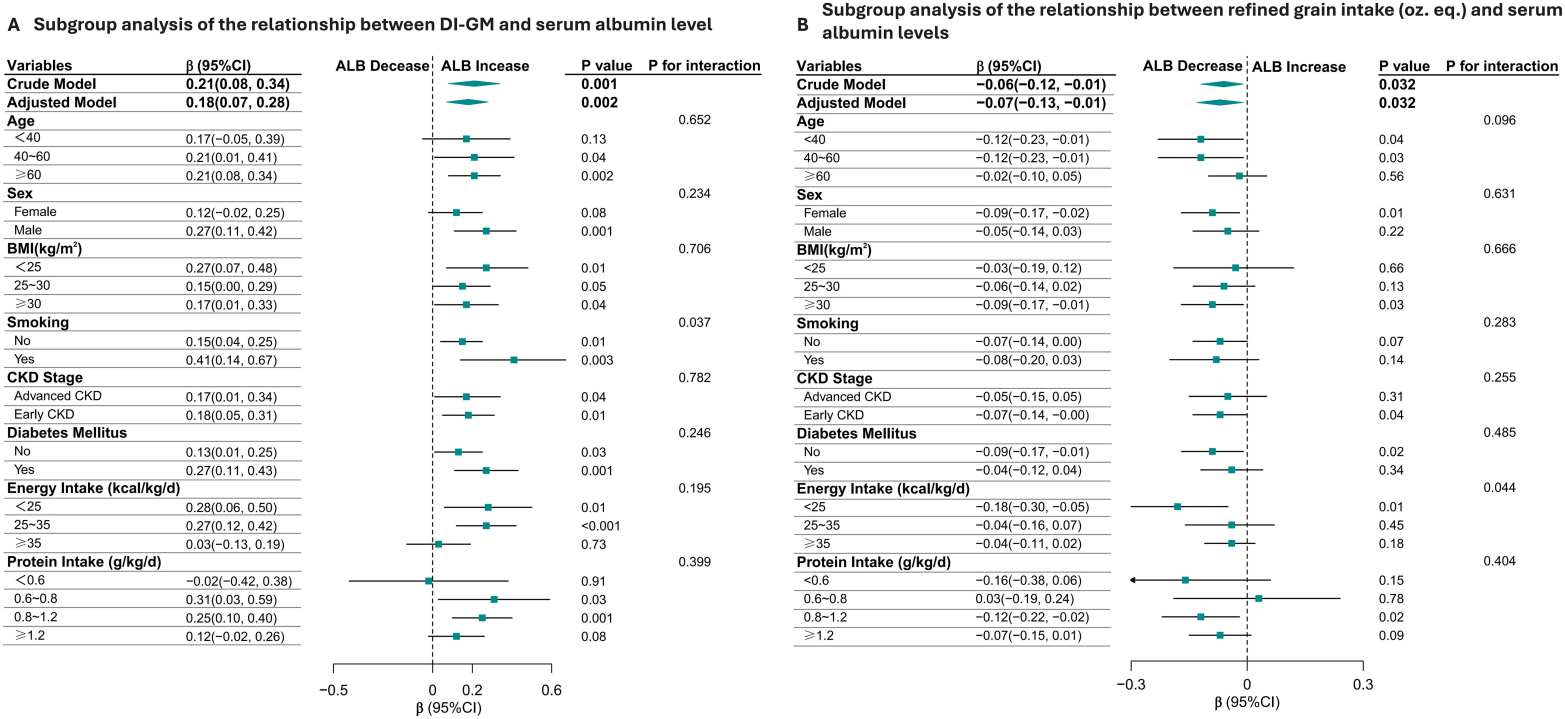
Subgroup Analysis of the relationship Between Dietary Index for Gut Microbiota (DI-GM), daily refined grain intake (oz.eq.) and Serum Albumin Levels in CKD Patients **(A, B)**. The positive association between DI-GM and serum albumin levels was consistent across most subgroups, with stronger effects observed in males, smokers, individuals with lower BMI, and those with diabetes. The significant interaction by smoking status suggests that smoking may modify the relationship between DI-GM and serum albumin **(A)**. Additionally, refined grain intake is consistently associated with lower serum albumin levels across most subgroups **(B)**.

The positive association between DI-GM and serum albumin levels was consistent across most subgroups, with stronger effects observed in males, smokers, individuals with lower BMI, and those with diabetes. The significant interaction by smoking status suggests that smoking may modify the relationship between DI-GM and serum albumin. Refined grains intake is consistently associated with lower serum albumin levels across most subgroups, with notable variations in the strength of the association depending on age, BMI, and sex. (**Figure 3**).

## Discussion

In this cross-sectional study, we explored the association between the DI-GM and serum albumin levels in adults with CKD. Our findings indicate a significant positive association between higher DI-GM scores and increased serum albumin levels, with a consistent reduction in the odds of hypoalbuminemia across all models. Additionally, refined grain intake emerged as a key adverse factor, showing a negative relationship with serum albumin levels. These results provide novel insights into the potential role of gut microbiota-targeted dietary interventions in CKD management.

The presence of hypoalbuminemia indicates a poor prognosis and worsened clinical outcomes in patients with CKD, cancer, and infections (23–27), due to its correlation with inflammatory and catabolic processes. Dysbiosis of the gut microbiota is widely reported in CKD (28–30) and has been associated with systemic inflammation, oxidative stress (31–33), and reduced nutrient absorption by nutritional management and complications control (34). These factors collectively contribute to hypoalbuminemia and poor clinical outcomes in CKD patients (35–38). Our study supports previous findings by demonstrating that a high DI-GM diet, rich in fiber, whole grains, and plant-based foods, is associated with improved serum albumin levels. The anti-inflammatory and alkalizing effects of plant-based diets may help mitigate CKD-associated acidosis (39, 40) and chronic inflammation (7, 41), which are critical contributors to hypoalbuminemia.

Among the components of the DI-GM score, refined grains emerged as a notable factor: a score of 1 for refined grains was associated with elevated serum albumin levels (crude model: β=0.56, (95%CI:0.13,0.98); adjusted model: β=0.41, (95%CI:0.02,0.80)). To confirm the robustness of the association between a refined grain score of 1 in the DI-GM and serum albumin, we extended our analysis to investigate daily refined grain intake as a continuous variable. Intriguingly, increased refined grain intake was associated with decreased serum albumin levels. Notably, this inverse association persisted in sensitivity analyses across crude models (β=-0.06, (95%CI: -0.12, -0.01)) and models adjusted for covariates (β=-0.07, (95%CI: -0.13,-0.01)). The negative association between refined grain intake and serum albumin levels highlights the potential harm of diets high in processed carbohydrates. Refined grains may exacerbate gut microbiota dysbiosis by promoting the growth of pathogenic bacteria, leading to increased production of uremic toxins and systemic inflammation. This finding aligns with studies suggesting that diets with a higher ratio of refined to whole grains are associated with CKD progression and worse clinical outcomes (42, 43).

Our findings suggest that the high DI-GM diet alleviates hypoalbuminemia in CKD patients probably through a multi-target mechanism involving gut microbiota-derived metabolites, with significant clinical implications for patient management. At the molecular level, Lactobacillus enrichment (e.g., L. johnsonii) enhances indole-3-aldehyde production to antagonize AhR-mediated renal inflammation and fibrosis (30, 44), while increased short chain fatty acids (SCFAs) (e.g., butyrate) (45) attenuate oxidative stress and NF-κB activation via histone deacetylase inhibition and G protein-coupled receptor109A activation (46). Concurrently, reduced uremic toxins (indoxyl sulfate) and endotoxins (lipopolysaccharide) preserve glomerular filtration barrier integrity by suppressing reactive oxygen species/transforming growth factor- β1 (ROS/TGF-β1) signaling (46). This mechanistic framework is substantiated by broader dietary intervention studies: plant-based diets rich in fiber promote SCFA-producing bacteria (e.g., Roseburia) with anti-inflammatory properties (13, 47–49), whereas red meat-heavy diets exacerbate CKD progression by elevating protein fermentation products (indoxyl sulfate, p-cresyl sulfate) and uremic toxins that intensify inflammation (50–52), while simultaneously decreasing gut microbiota diversity (50).

Clinically, high DI-GM diet implementation represents a practical intervention to improve serum albumin levels, thereby reducing the risk of complications such as protein-energy wasting and cardiovascular events. Most notably, our subgroup analyses revealed that the benefits of this dietary approach were more pronounced in specific high-risk populations—males, smokers, and individuals with diabetes (53, 54)—suggesting opportunities for personalized nutritional strategies. Collectively, these findings underscore that targeted dietary modulation of gut microbiota composition and metabolic output constitutes a critical strategy for mitigating proteinuria and hypoalbuminemia in CKD, highlighting the importance of promoting microbiota-friendly dietary patterns in comprehensive CKD management, particularly for vulnerable patient subgroups.

Furthermore, the lower DI-GM scores observed in non-Hispanic Black individuals and those with lower educational attainment underscore the need for culturally tailored dietary education and interventions. Addressing socioeconomic and racial disparities in dietary quality is essential for improving health outcomes in CKD populations. Culturally appropriate dietary strategies and accessible nutrition education programs should be prioritized to ensure equitable health benefits.

Our study also highlights the role of dietary quality over quantity. Although energy and protein intake did not differ significantly across DI-GM tertiles, higher-quality diets with increased fiber and whole grain intake were associated with improved serum albumin levels. These findings emphasize the importance of dietary composition and nutrient density in managing CKD-related complications.

Despite its strengths, including the use of a nationally representative sample and comprehensive adjustment for confounders, this study has several limitations. First, the cross-sectional design precludes causal inferences between DI-GM and serum albumin levels. Longitudinal studies are needed to confirm these associations and explore underlying mechanisms. Second, dietary data were self-reported, which may introduce recall bias or underreporting, particularly among older adults. Third, while we adjusted for a wide range of confounders, residual confounding cannot be entirely ruled out.

Future research should focus on validating the long-term effects of DI-GM-based diets on serum albumin levels and other clinical outcomes in CKD patients. Interventional studies are also needed to establish causality and assess the feasibility of implementing DI-GM diets in clinical practice. Additionally, exploring the molecular mechanisms underlying the observed associations, such as changes in gut microbiota diversity, SCFA production, and inflammatory pathways, could provide valuable insights into the therapeutic potential of gut-targeted dietary interventions.

In conclusion, a high DI-GM diet may provide a promising dietary approach for managing hypoalbuminemia in CKD patients by modulating gut microbiota composition and reducing systemic inflammation. These findings underscore the importance of personalized dietary interventions in CKD care and suggest potential avenues for future research aimed at optimizing dietary strategies to improve clinical outcomes in this vulnerable population. By prioritizing microbiota-friendly dietary practices, clinicians can improve patient outcomes and address the underlying inflammatory and nutritional challenges associated with CKD.

## Data Availability

The original contributions presented in the study are included in the article/**Supplementary Material**. Further inquiries can be directed to the corresponding author.

## Supplementary material

Supplementary Table 1 Components and scoring of the DI-GM (Source: Kase BE, et al. *Nutrients*. 2024;16(7):1045.).

Supplementary Table 2 Weighted Univariable and Multivariable Analysis of Dietary Components’ score in the Dietary Index for Gut Microbiota and Their Association with Serum Albumin Levels.
