# Supplementary material for: Microbiota-friendly diet ameliorates hypoalbuminemia in chronic kidney disease: evidence from NHANES
Source: Front Immunol. 2025 May 6;16:1546031. doi: 10.3389/fimmu.2025.1546031 (PMC12088941; doi:10.3389/fimmu.2025.1546031)
Supplement: Supplementary Table 1 — Components and scoring of the DI-GM (Source: Kase BE, et al. Nutrients. 2024;16(7):1045.). [file DataSheet1.docx]

| **Table S1. Components and scoring of the DI-GM (Source: Kase BE, et al. *Nutrients*. 2024;16(7):1045.)** | | |
| --- | --- | --- |
| **Component** | **Included Foods within the component** | **Scoring** |
| **Beneficial to gut microbiota** |  |  |
| Avocados | Avocados | For each component, a score of 1 if consumption at or above the sex-specific median ,else 0 |
| Broccoli | Broccoli |  |
| Chickpea | Chickpea |  |
| Coffee | Coffee |  |
| Cranberries | Cranberries |  |
| Fermented diary | Yogurt,cheese,kefir,sour cream,buttermilk |  |
| Fiber | Not applicable |  |
| Green tea | Green tea |  |
| Soybean | Soy products--Soy milk, Tofu |  |
| Whole grains | Grains defined as whole grains, containing the entire grain kernel-the bran,germ, and endosperm |  |
| **Unfavorable to gut microbiota** |  |  |
| **High-fat diet(% energy)** | **Not applicable** | 0 if consumption at or above 40% energy from fat,else 1.For each remaining component, a score of 0 if comsumption at or above the sex-specific median, else 1. |
| Processed meat | Frankfurters,sausages,corned beef, and luncheon meat that are made from beef, pork, or poultry |  |
| Red meat | Beef,veal,pork,lamb, and game meat; excludes organ meat and cured meat |  |
| Refined grains | Refined grains that do not contain all of the components of entire grain kernel |  |

| **Table S2.** **Univariable and Multivariable Analysis of Dietary Components' score in the Dietary Index for Gut Microbiota and Their Association with Serum Albumin Levels** | | | | |
| --- | --- | --- | --- | --- |
| **Estimated score of DI-GM and Components** | **Estimated serum albumin(g/L)** | | | |
|  | **Crude Model** | | **Adjusted Model** | |
|  | β(95%CI) | P value | β (95%CI) | P value |
| **Total DI-GM** | **0.21(0.08, 0.34)** | **0.001** | **0.18(0.07, 0.28)** | **0.002** |
| **Score of Avocados** |  |  |  |  |
| 0 | Reference |  | Reference |  |
| 1 | 0.23(-0.86, 1.33) | 0.673 | -0.19(-1.20, 0.82) | 0.709 |
| **Score of Broccoli** |  |  |  |  |
| 0 | Reference |  | Reference |  |
| 1 | 0.51(-0.08, 1.09) | 0.088 | 0.49(0.01, 0.96) | 0.709 |
| **Score of Chickpea** |  |  |  |  |
| 0 | Reference |  | Reference |  |
| 1 | 1.39(-0.03, 2.80) | 0.055 | 0.34(-1.32, 2.00) | 0.684 |
| **Score of Coffee** |  |  |  |  |
| 0 | Reference |  | Reference |  |
| 1 | 0.33(-0.06, 0.73) | 0.099 | 0.47(0.09, 0.84) | 0.016 |
| **Score of cranberries** |  |  |  |  |
| 0 | Reference |  | Reference |  |
| 1 | -0.1(-0.79, 0.58) | 0.765 | -0.13(-0.76, 0.51) | 0.687 |
| **Score of fermented dairy** |  |  |  |  |
| 0 | Reference |  | Reference |  |
| 1 | -0.02(-0.41, 0.38) | 0.934 | -0.1(-0.48, 0.27) | 0.588 |
| **Score of fiber** |  |  |  |  |
| 0 | Reference |  | Reference |  |
| 1 | 0.19(-0.15, 0.52) | 0.270 | 0.17(-0.17, 0.52) | 0.320 |
| **Score of green tea** |  |  |  |  |
| 0 | Reference |  | Reference |  |
| 1 | -0.2(-0.69, 0.29) | 0.418 | 0.05(-0.42, 0.51) | 0.840 |
| **Score of soybeans** |  |  |  |  |
| 0 | Reference |  | Reference |  |
| 1 | 0.48(-0.04, 1.00) | 0.070 | 0.35(-0.12, 0.83) | 0.142 |
| **Score of whole grains** |  |  |  |  |
| 0 | Reference |  | Reference |  |
| 1 | 0.51(0.02, 1.01) | 0.042 | 0.41(-0.04, 0.85) | 0.072 |
| **Score of refined grains** |  |  |  |  |
| 0 | Reference |  | Reference |  |
| 1 | **0.56(0.13, 0.98)** | **0.010** | **0.41(0.02, 0.80)** | **0.041** |
| **Score of processed meat** |  |  |  |  |
| 0 | Reference |  | Reference |  |
| 1 | 0.5(-0.03, 1.02) | 0.062 | 0.44(0.00, 0.88) | 0.048 |
| **Score of red meat** |  |  |  |  |
| 0 | Reference |  | Reference |  |
| 1 | 0.28(-0.10, 0.67) | 0.150 | 0.41(0.01, 0.82) | 0.042 |
| **Score of fat** |  |  |  |  |
| 0 | Reference |  | Reference |  |
| 1 | -0.18(-0.63, 0.27) | 0.437 | -0.4(-0.79, -0.01) | 0.045 |

**Adjusted Model：**Adjusted for Age + sex+ race + PIR+Education+Marital Status +BMI+Smoking status+Drinking status + eGFR+ urinary albumin +Energy intake(kcal/kg/d) +Protein intake(g/kg/d) + cardiovascular disease +Hyperlipidemia+Hypertension+ Diabetes Mellitus
